# Comparing the Folds of Prions and Other Pathogenic Amyloids

**DOI:** 10.3390/pathogens7020050

**Published:** 2018-05-04

**Authors:** José Miguel Flores-Fernández, Vineet Rathod, Holger Wille

**Affiliations:** Department of Biochemistry & Centre for Prions and Protein Folding Diseases, University of Alberta, 204 Brain and Aging Research Building, Edmonton, AB T6G 2M8, Canada; floresfe@ualberta.ca (J.M.F.-F.); vrathod@ualberta.ca (V.R.)

**Keywords:** prion structure, β-solenoid, PHF-tau structure, Aβ(1-42) fibril structure, α-synuclein amyloid structure, parallel in-register β-structure

## Abstract

Pathogenic amyloids are the main feature of several neurodegenerative disorders, such as Creutzfeldt–Jakob disease, Alzheimer’s disease, and Parkinson’s disease. High resolution structures of tau paired helical filaments (PHFs), amyloid-β(1-42) (Aβ(1-42)) fibrils, and α-synuclein fibrils were recently reported using cryo-electron microscopy. A high-resolution structure for the infectious prion protein, PrP^Sc^, is not yet available due to its insolubility and its propensity to aggregate, but cryo-electron microscopy, X-ray fiber diffraction, and other approaches have defined the overall architecture of PrP^Sc^ as a 4-rung β-solenoid. Thus, the structure of PrP^Sc^ must have a high similarity to that of the fungal prion HET-s, which is part of the fungal heterokaryon incompatibility system and contains a 2-rung β-solenoid. This review compares the structures of tau PHFs, Aβ(1-42), and α-synuclein fibrils, where the β-strands of each molecule stack on top of each other in a parallel in-register arrangement, with the β-solenoid folds of HET-s and PrP^Sc^.

## 1. Introduction

Pathogenic amyloids are the hallmark of many neurodegenerative diseases, such as Creutzfeldt-Jakob disease (CJD) [1], Alzheimer’s disease (AD) [2], and Parkinson’s disease (PD) [3]. Most proteins are subjected to covalent or non-covalent post-translational modifications, including N-linked glycosylation, disulfide bond formation, and protein folding, during which the protein obtains its native conformation and its functional state. Proper folding allows for the protein to reach and maintain a stable state and to exert its biological function(s). However, the amyloid state results from a process by which monomeric proteins or poorly folded peptides self-assemble into fibrillar aggregates, termed “amyloid”. Amyloid fibrils have a common architecture, consisting of a “cross-β structure”, which is independent of the native fold of the protein [4]. The misfolded proteins for CJD, AD, and PD are PrP^Sc^, amyloid-β and microtubule-associated protein tau (tau), and α-synuclein, respectively. These proteins misfold and aggregate into a very stable β-sheet architecture, and eventually form amyloid fibrils. The molecular mechanisms how the altered conformations of these disease-associated proteins lead to slow, progressive neurodegeneration remain elusive. Recent studies on the spread of toxic forms of the tau protein and of truncated Aβ, which results from the proteolytic cleavage of the amyloid precursor protein, indicated that they share characteristics with PrP^Sc^ [5]. To date, there is no effective treatment for the prion diseases and most other pathogenic amyloids [6], thus making it necessary to understand the structure, formation, and aggregation processes of these pathogenic proteins to devise therapeutic strategies.

The infectious prion protein, termed PrP^Sc^, has the ability to convert native PrP^C^ into a copy of itself, adopting a non-native conformation that has the propensity to self-assemble into amyloid fibrils [7]. The main features that distinguish PrP^Sc^ from PrP^C^ are its high content in β-structure [8], its partial resistance to proteases [9], its insolubility, and its propensity to aggregate into amyloid fibrils and other quaternary structures [10], which accumulate over time, resulting in brain cell and tissue damage [11]. Based on its structural properties, PrP^Sc^ differs in a number of epitopes from those that are recognized by antibodies targeting PrP^C^ [12]. Over the years, many structural models for PrP^Sc^ have been proposed [11]; however, the most prominent ones are the β-solenoid [13], the β-spiral [14], and the parallel in-register β-sheet models [15]. The insoluble nature and the propensity of PrP^Sc^ to aggregate make it difficult to determine its structure. Hence, the high-resolution structure of PrP^Sc^ is still unknown, although efforts have been made to gain insights by combining techniques, such as X-ray fiber diffraction [16], electron microscopy (EM) [17], and limited proteolysis using proteinase K [18]. Together, these data indicated that PrP^Sc^ is a β-solenoid protein, consisting of 4-stacked β-rungs [7]. However, it is still unknown whether the β-solenoid twists in a left- or right-handed sense.

Recent insights into the structure of PrP^Sc^ have revealed similarities and differences with the structures of non-pathogenic proteins adopting a β-solenoid fold. Moreover, other classes of pathogenic amyloids, such as Aβ(1-42) fibrils, tau PHFs, and α-synuclein fibrils, which have been recently solved by solid-state NMR spectroscopy (ssNMR) and cryo-EM [19,20,21,22,23], provide novel insights into the folds of self-propagating amyloids that cause neurodegenerative diseases. While these last three amyloids do not fall under the criteria of a β-solenoid, they are similar enough to warrant a side-by-side comparison. Thus, this review provides a comparison between the folds of PrP^Sc^ and those of other pathogenic amyloids.

### 2. β-solenoid Amyloids: PrP^Sc^ and HET-s

#### 2.1. The Structure of PrP^Sc^

The current knowledge on the structure of PrP^Sc^ has been summarized in another review of this special issue [7]. Therefore, we provide only a short overview on what is known about the structure of PrP^Sc^, its proteolytically truncated variant (PrP27-30), and related molecular species.

**Figure 1:**
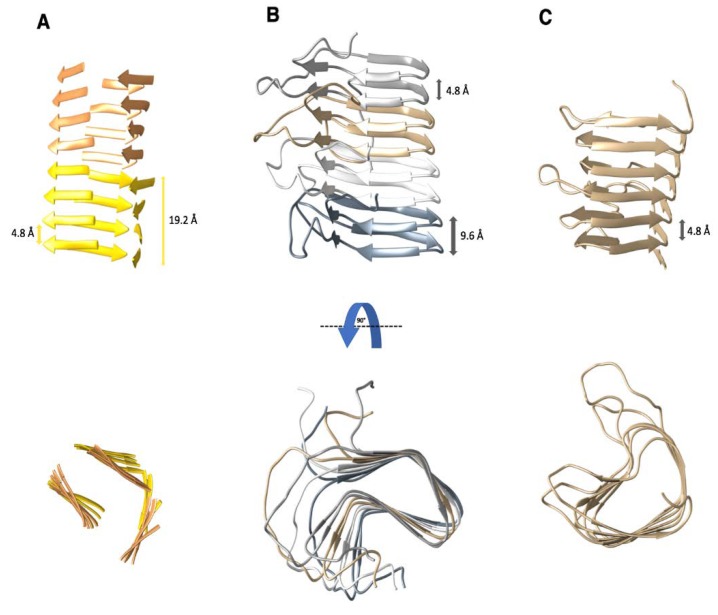
Three examples of proteins that adopt a β-solenoid fold. The characteristic distance between individual β-rungs is 4.8 ± 0.2 Å. (**A**) Representative model of the 4-rung β-solenoid architecture of PrP^Sc^ based on X-ray fiber diffraction and cryo-EM [16,26]. Characteristic distances of the 4-rung β-solenoid spacing are labeled. Each cartoon color represents a single PrP^Sc^ monomer. (**B**) Structure of an amyloid fibril formed by the prion domain (residues 218–289) of the fungal prion HET-s. Each monomer adopted a 2-rung left-handed β-solenoid fold and is shown in a different color. PDB access code: 2rnm [31]. (**C**) Structure of the right-handed β-solenoid protein pectate lyase C from *Erwinia chrysanthemi*. Its N- and C-terminal caps were removed for representation of the β-solenoid structure (residues 118–285). PDB access code: 2pec [32].

Fourier-transform infrared (FTIR) spectroscopy provided the first experimental evidence that the N-terminally truncated PrP27-30 contains predominantly β-structure [8,24]. Electron crystallography analyses on 2D crystals of PrP27-30 and an engineered variant of only 106 residues, PrP^Sc^106, suggested the presence of a β-solenoid fold as a key feature of the infectious conformer [13,17]. Subsequently, X-ray fiber diffraction determined the molecular height of PrP27-30 in amyloid fibrils to be 19.2 Å, corresponding to the height of 4 β-strands (19.2 Å = 4 × 4.8 Å) [16]. In addition, the diffraction data confirmed that the core of PrP27-30 adopts a β-solenoid fold, consisting of 4-stacked β-rungs (Figure 1A). The repeating unit size of 19.2 Å was also found in the diffraction patterns that were obtained from PrP^Sc^106 amyloid fibrils [25]. Recently, cryo-EM and subsequent three-dimensional (3D) reconstructions demonstrated that PrP27-30 amyloid fibrils can be formed by two intertwined protofilaments. Furthermore, the cryo-EM analysis corroborated that the structure of PrP27-30 consists of a 4-rung β-solenoid [26].

PrP^Sc^ has a β-sheet core that is assumed to be water-inaccessible with individual β-strands that are connected by short turns and loops [27], although it is still unknown which residues are in each β-strand and which ones are facing outward or inward with respect to the β-solenoid core [7,28]. A proposed model, based on mass spectrometry of proteinase K-resistant fragments obtained from PrP27-30, suggests which amino acids may be located in β-strands or connecting loops [18,28]. The structure of PrP^Sc^ has a high degree of stability, which provides resistance against denaturation and decontamination, but not all of the factors contributing to this stability are fully understood. It is clear that the highly-ordered hydrogen bonds that run up and down the β-sheets are essential for the structure and stability of the PrP^Sc^ β-solenoid fold [27]. However, van-der-Waals forces, hydrophobic and electrostatic interactions, as well as aromatic side-chain stacking also contribute to its stability, as it has been demonstrated in other amyloids [29] and β-solenoid proteins [30].

#### 2.2. The Structure of the HET-s Prion Domain

HET-s is a known functional prion of the filamentous fungus *Podospora anserina* and it is involved in regulating heterokaryon incompatibility among different mating types [33,34]. The prion domain of HET-s (HET-s(218-289)) is able to form amyloid fibrils and it becomes protease resistant in the process [35]. Its structure, which has been solved by ssNMR, consists of a left-handed, 2-rung β-solenoid (Figure 1B) [31,36]. The β-solenoid rungs are connected by a flexible loop of 15 residues [37]. There are eight β-strands per molecule, and each β-solenoid rung has four β-strands connected by short loops. The first two β-strands are connected by a short, 2-residue β-arc, changing the orientation of the peptide backbone by ~90°; the second and third β-strands are connected by a 3-residue β-arc, changing the orientation by ~150°, and, lastly, the third and fourth β-strands are connected by a single glycine residue [31].

HET-s(218-289) has a triangular hydrophobic core formed by the first three β-strands of each β-solenoid rung, which also includes two buried polar amino acids (T233 and S273) and two asparagine ladders consisting of N226/N262 and N243/N279 [31]. The fourth β-strand is pointing away from the β-solenoid core, forming part of the loop that connects the first and the second rungs of the β-solenoid structure. The β-strands of the second β-solenoid rung stack on top of the β-strands of the first rung, forming four intramolecular β-sheets. These β-sheets can connect with the β-strands of the next HET-s(218-289) molecule through intermolecular hydrogen bonds/β-sheet contacts [31]. HET-s(218-289) contains polar and charged amino acids that are exposed on the surface of the β-solenoid structure, where three salt bridges are formed between the residues K229, E234 and R236 from the first β-rung and the residues E265, K270, and E272 from the second β-rung [36]. The HET-s(1-227) N-terminal domain consists of nine α-helices and two short β-strands [38,39]. Overall, HET-s(218-289) has an amino acid composition that is very different to the yeast prions which are Q/N rich [40].

In addition to the characterization via ssNMR, HET-s(218-289) has also been analyzed by cryo-EM [41] and X-ray fiber diffraction [42,43]. In the latter studies, meridional reflections at ~4.8 Å and ~9.6 Å confirmed that HET-s(218-289) adopts a 2-rung β-solenoid structure with clear similarities to the structure of PrP^Sc^ [16,42].

## 3. The β-Solenoid Fold of Non-Pathogenic Proteins

β-solenoid proteins are characterized by a polypeptide chain that folds into more or less regular “solenoidal windings” (Figure 1B,C), while the canonical β-helical proteins follow a more stringent helical geometry [44]. β-solenoid proteins contain between three to well above 100 β-rungs [45]. Each β-rung contains two to four β-strands and they are connected by tight turns, β-arcs (two to six residues), or longer loops (Figure 1C). Overall, the β-rungs have a length between 12 and 30 amino acids. A β-rung corresponds to a complete turn of the amino acid backbone to where the next β-rung begins with an axial rise of 4.8 ± 0.2 Å [44,46]. The β-rungs that form the β-solenoid structure are connected by hydrogen bonds to the β-rungs above and below, forming a hydrophobic core with solvent-exposed side-chains on the surface [44,47]. A distinctive feature of β-solenoid proteins is the stacking of identical residues on the same position in subsequent β-rungs [44,48]. Such “ladders” are usually comprised of polar residues, with asparagine being the most commonly followed by serine and threonine, but aromatic residues can also form separate ladders that are stabilized by aromatic stacking [44,47,49,50].

β-solenoid proteins can be classified into right- or left-handed polymers, depending on the direction in which the polypeptide chain winds around the axis [30]. In addition, β-solenoid proteins can display a twist, which is determined by an angular offset between individual β-rungs [30,49]. The shape of a β-solenoid cross-section is defined by the β-arcs connecting each β-strand, with the most frequent shapes being generally triangular, rectangular, or oval [44]. The short β-arcs that connect individual β-strands are mainly formed by non-polar and uncharged polar residues [46], but longer loops can also connect subsequent β-rungs, while retaining the overall shape of the β-solenoid. Lastly, the N- and C-termini of β-solenoid proteins generally have polymerization-inhibiting caps, which contain polar and charged amino acids and protect the hydrophobic core from solvent exposure [51,52].

## 4. Amyloid Folds of Other Pathogenic Proteins

### 4.1. Short Amyloid Peptides

Short, amyloidogenic peptides can be studied in great detail due to their small size, propensity to form highly regular amyloid structures, and straightforward availability through synthetic routes. Thus, short peptides of the prion protein have been analyzed as microcrystals that were obtained from amyloid fibril preparations [53,54], while the structure of the more complex, full-length PrP^Sc^ remains unsolved [7].

These peptide structures have a minimal cross-β-sheet structure, and the structural motif was named a “steric zipper”, based on the interdigitation of the side chains from neighboring β-sheets. In total, eight classes of steric zippers were described for these short amyloid peptides [55]. The steric zipper classes are defined by the orientation and the stacking of the β-strands that make up individual protofilaments—running either in parallel or antiparallel orientation. Moreover, adjacent β-sheets may pack face-to-face or face-to-back, and they also could be oriented up-up or up-down [54]. Examples of peptides that are derived from human (SNQNNF) or elk prion proteins (NNQNTF) adopted different steric zipper conformations [54,56], indicating the structural variability that is accessible to such short peptides. It was speculated that these sequence stretches would adopt similar conformations in PrP^Sc^, but the X-ray fiber diffraction data contained no indication of such tight stacking of β-sheets in PrP^Sc^/PrP27-30 [16,25]. Similarly, the tau peptide VQIVYK [54] does not adopt the same steric zipper arrangement in the PHF-tau structure that was recently elucidated by cryo-EM [20] (see below).

### 4.2. The Structure of PHF-Tau

The microtubule-associated protein tau is a natively unfolded protein consisting of up to 441 amino acids. It plays a key role in maintaining the elongated morphology of neurons by stabilizing microtubules in the axon [57,58]. The tau protein contains three or four sequence repeats (R1, R2, R3, and R4) as part of the microtubule-binding domain, with the presence of the repeat R2 being determined by alternative splicing of the mRNA. Tau inclusions are observed in the brains of diseased patients, either as paired helical filaments (PHFs) or straight filaments (SFs), with similar protofilament structures [5,59,60].

**Figure 2:**
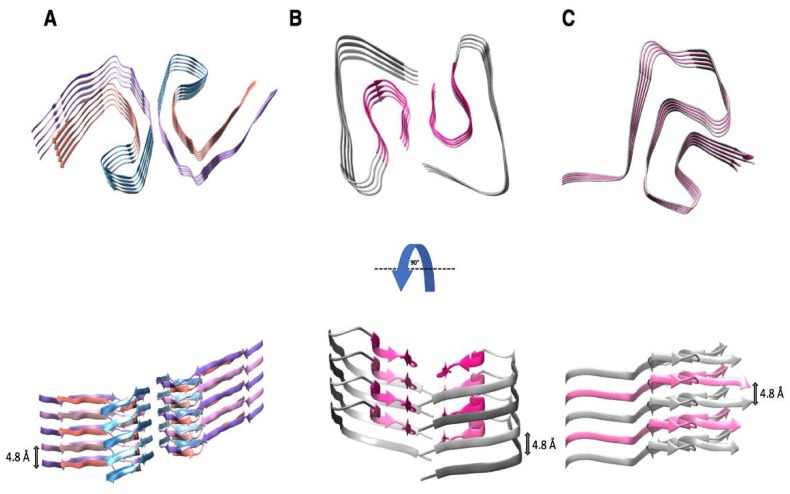
Structures of pathogenic amyloid fibrils (**A**) paired helical filament (PHF) of tau; (**B**) Aß(1-42); and, (**C**) α-synuclein. The fibril cores of these proteins are arranged as parallel in-register ß-sheet structures, which are stabilized by hydrophobic interactions, salt bridges, and hydrogen bonds up and down the fibril axis. The axial distance between the stacked protein molecules is 4.8 ± 0.2 Å. (**A**) Top and the side views of a high-resolution structure of PHFs obtained by cryo-EM [20]. Five successive layers of the tau protein along the fibril axis revealed that the fibril core is composed of two C-shaped subunits (residues 306–378). PDB access code: 5o3l [20]. (**B**) Top and side views of a high-resolution structure of an Aß(1-42) fibril produced by cryo-EM [19]. The LS-shaped cross-sections of each protofilament reveal a staggered stacking of molecules along the fibril axis. PDB access code: 5oqv [19]. (**C**) Top and side views of a high-resolution structure of α-synuclein determined by ssNMR and X-ray fiber diffraction [21]. The fibril core of α -synuclein contains a Greek key motif based on a parallel in-register ß-sheet topology (residues 42–96). PDB access code: 2n0a [21].

Studies involving X-ray fiber diffraction, circular dichroism (CD) spectroscopy, and FTIR spectroscopy on PHFs and SFs revealed a common cross-β architecture with stacks of β-strands being arranged perpendicular to the fiber axis [61,62,63]. A recent, high-resolution cryo-EM structure of PHFs and SFs at a resolution of 3.4 Å showed that R3 and R4 (residues V306–F378) form the core of intertwined protofilaments that are based on a C-shaped subunit (Figure 2A) [20]. The protofilament cores in PHFs and SFs are similar in structure and contain 8 β-strands that form the C-shaped subunit by packing in an overall antiparallel arrangement [20]. The organization of these β-strands is highly ordered through parallel in-register stacking of tau molecules, allowing for identical amino acids to stack on top of one another and forming hydrogen bond networks along the fiber axis with a helical rise of 4.8 Å [20,64]. The first β-strand of the PHF core contains the hexapeptide _306_VQIVYK_311_, which aligns through face-to-face packing with the residues _373_THKLTF_378_ of the eighth β-strand [20]. The interaction is based on hydrophobic contacts that are similar to a steric zipper, but distinct from the homotypic VQIVYK steric zipper that was predicted earlier by analyzing short, amyloidogenic peptides only [54].

An interesting feature that was observed in the structure of PHFs is a β-helix-like structure spanning residues 337–353 in R3, which resembles a single β-solenoid rung of the HET-s prion domain [20,31]. Thus, the core of PHFs contains two structural motifs parallel in-register β-sheets and β-helices. Overall, the side chains of the amino acids facing the exterior of the PHFs help to increase the stability by forming hydrogen bonds between charged residues, asparagines, and glutamines, while the β-helix-like structure and the interior of the PHF protofilaments are stabilized by hydrophobic clustering and aliphatic and aromatic stacking [20,55].

### 4.3. The Structure of Aβ(1-42)

Aβ is a peptide that is released through proteolytic cleavage of the amyloid precursor protein (APP), which is expressed in many tissues, including neurons [5,65]. It has been shown that the Aβ C-terminal variants Aβ(1-40) and Aβ(1-42) are prone to aggregation, forming toxic oligomers and amyloid fibrils. Aβ(1-42) is the predominant Aβ molecule that is found in neuritic plaques of Alzheimer’s disease patients [65,66], but it is unclear how Aβ aggregates damage the brain and give rise to neuronal dysfunction and cellular toxicity [67].

Among others, studies using X-ray fiber diffraction, EM, and ssNMR have shown that Aβ fibrils consist of two protofilaments that intertwine along the fibril axis, suggesting a two-fold symmetry [67,68]. The fibrils were observed to have protofilaments with a diameter of ~5 nm and a ~110 nm cross over distance with a left-handed twist [65,69,70]. ssNMR indicated that each Aβ molecule contains four β-strands between amino acids 15 to 42, resulting in a highly ordered structure at the C-terminus of the peptide [55]. Within each Aβ molecule, the four β-strands stack on top of each other, forming an S-shaped structure (Figure 2B). The individual β-strands were stabilized by hydrogen bonds to the molecules above and below, forming contiguous β-sheets that run parallel to the fibril axis. Moreover, the C-terminus of each S-shaped Aβ molecule formed the fibril core with residues 23 to 26 interacting in a steric zipper-like fashion [55,67].

More recently, a high-resolution cryo-EM structure revealed that Aβ(1-42) fibrils do not possess a two-fold symmetry, but instead adopt a screw symmetry with a rise of ~4.8 Å. The staggered arrangement, which produced the screw symmetry and cannot be detected via ssNMR, resulted in each molecule of Aβ(1-42) interacting with six other molecules in the minimal fibril unit. Moreover, as a consequence of the screw symmetry, the Aβ(1-42) fibrils possess distinct end surfaces on the two protofilaments, referred to as “groove” and “ridge”, based on residues 27–33, which either formed a protrusion or indentation at the protofilament end [19]. In addition, the Aβ(1-42) fibril structure was stabilized by salt bridges between residues D1 and K28, D7 and R5, as well as E11 and H6 / H13. The hydrophobic residues were buried in the protofilament core, while the polar side chains were facing the outside of the protofilaments [19].

### 4.4. The Structure α-Synuclein

Relatively little is known about the structure and the function of the cytoplasmic protein, α-synuclein, which is mainly found in the presynaptic terminals of neuronal cells. The native form consists of three domains namely: the N-terminal lipid-binding α-helix (residues 1–60), the amyloid binding central domain (NAC; residues 61–95), and the C-terminal acidic tail (residues 96–140) [21,71,72]. α-synuclein is a natively unfolded protein in aqueous solution and an α-helical protein in association with phospholipids. Monomers of α-synuclein tend to aggregate into oligomers through an unknown mechanism, which then further assemble forming long amyloid fibrils. These fibrils are seen in the Lewy bodies and Lewy neurites from patients with Parkinson’s disease (PD) [73]. A 3D structure of α-synuclein amyloid fibrils has been determined using ssNMR and was further validated by EM and X-ray fiber diffraction [21,74]. The ssNMR analysis showed that the core of the α-synuclein fibril consists of residues 44 to 97, forming seven β-strands that adopt a Greek-key topology [21,75]. The resulting β-sheets form a parallel in register cross-β structure that is stabilized by hydrogen bonds along the fibril axis.

Recently, a high-resolution structure of the full-length α-synuclein amyloid fibril was deciphered using cryo-EM at 3.4 Å resolution, which revealed two intertwined protofilaments that were composed of a staggered arrangement of subunits [23]. The fibrils adopt a 2_1_ symmetry similar to the one seen with PHF and Aβ(1-42) fibrils [19,20,23]. The fibril core consisted of residues 42 to 95 (as shown in Figure 2C) and contained 8 β-strands forming a parallel in-register β-sheet structure with a spacing of ~4.8 Å between the β-sheets [23]. The side chains in the core were tightly packed through hydrophobic and aromatic interactions, forming a hydrophobic pocket containing residues I88, A91, and F94 [21,76]. The observed Greek-key topology was supported through strong hydrophobic interactions between residues V77, V82, A89, and A90, which formed β-strands [21]. Moreover, the β-strands in each molecule of α-synuclein were supported by a glutamine ladder along the fibril axis and turns that mainly consisted of small hydrophobic amino acids, such as alanine and glycine.

## 5. Similarities and Differences between Common Amyloid Folds

Many different techniques have been used to elucidate the structures of amyloid fibrils from neurodegenerative diseases as well as functional amyloids [77]. These structures have been studied using recombinant proteins, short peptides, as well as full-length proteins (see above). Substantial progress has been made using the classic X-ray fiber diffraction approach, as well as more recent techniques, such as ssNMR and cryo-EM [78]. A spate of structural studies on amyloids containing Aβ(1-42), α-synuclein, the tau protein (as PHFs and SFs), HET-s, and PrP^Sc^ revealed two predominant folds: the parallel in-register β-structure and the β-solenoid structure (compare Table 1).

### 5.1. Similarities between PrP^Sc^, HET-s, and β-solenoid Proteins

Interestingly, the two β-solenoid structures among the amyloids are both linked to prions and the autocatalytic conversion from an innocuous precursor into the prion state. Thus, it is informative to compare these two structures (to the degree that they are known) and those of other, non-pathogenic β-solenoid proteins. The HET-s(218-289) prion domain contains 71 residues, and its structure forms a 2-rung β-solenoid [31], which means that for a similar 4-rung β-solenoid structure, 142 residues would be needed. Intriguingly, this matches perfectly with the ~143 residues in the proteinase K-resistant core of PrP^Sc^ (PrP27-30), which was revealed to contain a 4-rung β-solenoid structure (Figure 1A,B) [7,16,26].

Many β-solenoids proteins contain asparagine ladders as a prominent surface feature, which contributes to the stability of the fold. This feature can also be found in the HET-s(218-289) 2-rung β-solenoid structure with N226/N262 and N243/N279 stacking on top of one another [31]. Other amino acids that are known to form stabilizing ladders on the surface of β-solenoids proteins are threonine and tyrosine, while glycine is prominently found in the turns that are connecting β-strands due to its small size [44,46,47]. The high proportion of these residues in sequence of PrP27-30 (Thr (7.7%), Tyr (7.7%) and Asn (6.3%)) suggests that such ladders could also stabilize the 4-rung β-solenoid fold of PrP^Sc^. In addition, PrP27-30 contains even more Gly (9.9%), which could serve to make tight turns between adjacent β-strands [28]. Moreover, β-solenoid proteins usually have a hydrophobic core that stabilizes its structure, while the side chains of solvent exposed residues tend to be hydrophilic [44,46,47]. This arrangement is also found in the structure of HET-s(218-289) [31], and are assumed to apply for the structure of PrP^Sc^ as well [7,11,28].

Lastly, most β-solenoid proteins have a cap at the N- or C-termini (or both), which prevents the propagation of the β-solenoid fold into other regions of the protein [51]. This cap also prevents β-solenoid proteins from aggregating, since the removal of the cap has been observed to result in insoluble and/or amyloidogenic proteins [79]. Since PrP^Sc^ has not been subjected to evolutionary selection it does not appear to contain a capping structure, which would prevent aggregation and the conversion of other protein molecules (e.g., PrP^C^) [7]. It could be argued that the N-terminal, α-helix rich domain of full-length HET-s could be considered a cap for the β-solenoid structure of the prion domain [39].

### 5.2. Comparisons of Aβ(1-42), α-Synuclein, PHF-tau and β-Solenoid Amyloids

Recent high-resolution structures for Aβ(1-42) and α-synuclein amyloid fibrils, as well as tau PHFs that were obtained via cryo-EM, demonstrated that all these fibrils are based on a parallel in-register β-sheet architecture [19,20,23]. This means that each successive layer in the fibril consists of another protein molecule that stacks on top of the preceding one without substantial translation or rotation. However, in all three pathogenic fibrils a staggered conformation of the subunits was observed in the structures determined by cryo-EM, which resulted in a slight tilt for each layer. In turn, this tilt imparted the fibrils with a 2_1_ screw symmetry. The parallel in-register stacking of proteins renders these assemblies very sensitive to charge repulsion wherever charged amino acids are found, unless salt bridges neutralize the charge imbalance.

Similarly, the amino acid ladders that can be found in β-solenoid proteins take advantage of stabilizing interactions between identical/similar residues (see above). Furthermore, charge repulsion between charged residues also threatens the stability of β-solenoid proteins, and needs to be overcome through formation of salt bridges. Nevertheless, the stacking of subsequent β-solenoid rungs allows for sequence variations that are not open to the parallel in-register β-sheet architecture. In contrast to the parallel in-register β-sheet fold, β-solenoid proteins possess a core that is encircled by a continuous peptide chain, and is mainly composed of hydrophobic residues, and which is formed by the inward facing amino acid side chains. The β-structure of the β-rungs causes the side chains of the amino acids to alternate between inward and outward facing orientations, respectively.

Thus, the structures of parallel in-register β-sheet amyloids and those of β-solenoid amyloids are governed by the same types of interactions, but they fall into separate structure classes. Nevertheless, substantial overlap exists and both HET-s(218-289) and the mammalian prion protein (PrP) can also adopt a parallel in-register fold, as demonstrated through X-ray fiber diffraction and ssNMR [16,42,80,81].

## 6. Conclusions

In the last few years, high-resolution structures have been determined from Aβ(1-42) fibrils [19,22], PHF-tau filaments [20], α-synuclein fibrils [21,23], as well as HET-s(218-289) [31,36,41] using cryo-EM and ssNMR. However, a high-resolution structure of PrP^Sc^ is still lacking [7], and only lower-resolution data are available [8,13,16,17,18,24,26,27]. Nevertheless, we can classify the structures of these amyloid fibrils as either a parallel in-register fold (Aβ(1-42), PHF-tau, and α-synuclein) or a β-solenoid fold (HET-s(218-289) and PrP^Sc^). While these folds differ in their principle architecture, they have many molecular details in common (compare Table 1). In addition, the pathogenic amyloids are well known for their resistance against denaturation, proteolysis, and biological clearance, which is based on the properties of the underlying amyloid fold. A detailed understanding how these molecular details influence the pathogenicity of the protein fibrils/aggregates is still missing.

## Figures and Tables

**Table 1:** Comparison of structural features in pathogenic and non-pathogenic amyloids.

	Protein/Protein Aggregate	β-Solenoid	Parallel in-Register	Steric Zippers		Salt Bridges ^2^		Hydro-Phobic Core	Symmetry of Proto-Filaments
				Homo-Steric ^1^	Hetero-Steric ^2^	Intra-Molecular	Inter-Molecular		
**Pathogenic proteins**	**PHF-tau**	**(partial)**	**+**	**1**	**6**	**5**	**2**	**+**	**2_1_ screw**
	**Aβ(1-42)**	−	**+**	**1**	**2**	**0**	**2**	**+**	**2_1_ screw**
	**α-synuclein**	−	**+**	**1**	**2**	**2**	**2**	**+**	**2_1_ screw**
	**PrP^Sc^**	**+**	−	**0**	**0**	**unknown**	**unknown**	**+**	**unknown**
**Non-pathogenic proteins**	**HET-s**	**+**	−	**0**	**0**	**3**	**0**	**+**	**N. A.**
	**Pectate lyase C**	**+**	−	**0**	**0**	**15**	**N. A.**	**+**	**N. A.**

^1^ observed at the protofilament interface; ^2^ based on two protofilaments.

## References

[1] Gibbs C.J.J., Gajdusek D.C., Asher D.M., Alpers M.P., Beck E., Daniel P.M., Matthews W.B. (1968). Creutzfeldt-Jakob Disease (Spongiform Encephalopathy): Transmission to Chimpanzee. Science.

[2] Moller H.J., Graeber M.B. (1998). The case described by Alois Alzheimer in 1911. Eur. Arch. Psychiatry Clin. Neurosci..

[3] Parkinson J. (1817). An Essay on the Shaking Palsy.

[4] Eisenberg D., Jucker M. (2012). The amyloid state of proteins in human diseases. Cell.

[5] Tatarnikova O.G., Orlov M.A., Bobkova N.V. (2015). Beta-Amyloid and Tau-Protein: Structure, Interaction, and Prion-Like Properties. Biochemistry.

[6] Giles K., Olson S.H., Prusiner S.B. (2017). Developing Therapeutics for PrP Prion Diseases. Cold Spring Harb. Perspect. Med..

[7] Wille H., Requena J.R. (2018). The Structure of PrPSc Prions. Pathogens.

[8] Caughey B.W., Dong A., Bhat K.S., Ernst D., Hayes S.F., Caughey W.S. (1991). Secondary structure analysis of the scrapie-associated protein PrP 27-30 in water by infrared spectroscopy. Biochemistry.

[9] Prusiner S.B., Bolton D.C., Groth D.F., Bowman K.A., Cochran S.P., McKinley M.P. (1982). Further purification and characterization of scrapie prions. Biochemistry.

[10] Vázquez-Fernández E., Young H.S., Requena J.R., Wille H. (2017). The Structure of Mammalian Prions and Their Aggregates. Int. Rev. Cell Mol. Biol..

[11] Requena J.R., Wille H. (2014). The Structure of the Infectious Prion Protein. Prion.

[12] Horiuchi M., Karino A., Furuoka H., Ishiguro N., Kimura K., Shinagawa M. (2009). Generation of Monoclonal Antibody That Distinguishes PrPSc from PrPC and Neutralizes Prion Infectivity. Virology.

[13] Govaerts C., Wille H., Prusiner S.B., Cohen F.E. (2004). Evidence for Assembly of Prions with Left-Handed-Helices into Trimers. Proc. Natl. Acad. Sci. USA.

[14] DeMarco M.L., Daggett V. (2004). From Conversion to Aggregation: Protofibril Formation of the Prion Protein. Proc. Natl. Acad. Sci. USA.

[15] Cobb N.J., Sönnichsen F.D., Mchaourab H., Surewicz W.K. (2007). Molecular Architecture of Human Prion Protein Amyloid: A Parallel, in-Register β-Structure. Proc. Natl. Acad. Sci. USA.

[16] Wille H., Bian W., McDonald M., Kendall A., Colby D.W., Bloch L., Ollesch J., Borovinskiy A.L., Cohen F.E., Prusiner S.B. (2009). Natural and Synthetic Prion Structure from X-Ray Fiber Diffraction. Proc. Natl. Acad. Sci. USA.

[17] Wille H., Michelitsch M.D., Guenebaut V., Supattapone S., Serban A., Cohen F.E., Agard D.A., Prusiner S.B. (2002). Structural Studies of the Scrapie Prion Protein by Electron Crystallography. Proc. Natl. Acad. Sci. USA.

[18] Vázquez-Fernández E., Alonso J., Pastrana M.A., Ramos A., Stitz L., Vidal E., Dynin I., Petsch B., Silva C.J., Requena J.R. (2012). Structural Organization of Mammalian Prions as Probed by Limited Proteolysis. PLoS ONE.

[19] Gremer L., Schölzel D., Schenk C., Reinartz E., Labahn J., Ravelli R.B.G., Tusche M., Lopez-Iglesias C., Hoyer W., Heise H. (2017). Fibril Structure of Amyloid-β(1–42) by Cryoelectron Microscopy. Science.

[20] Fitzpatrick A.W.P., Falcon B., He S., Murzin A.G., Murshudov G., Garringer H.J., Crowther R.A., Ghetti B., Goedert M., Scheres S.H.W. (2017). Cryo-EM Structures of Tau Filaments from Alzheimer’s Disease. Nature.

[21] Tuttle M.D., Comellas G., Nieuwkoop A.J., Covell D.J., Berthold D.A., Kloepper K.D., Courtney J.M., Kim J.K., Barclay A.M., Kendall A. (2016). Solid-State NMR Structure of a Pathogenic Fibril of Full-Length Human α-Synuclein. Nat. Struct. Mol. Biol..

[22] Wälti M.A., Ravotti F., Arai H., Glabe C.G., Wall J.S.4, Böckmann A., Güntert P., Meier B.H., Riek R. (2016). Atomic-resolution structure of a disease-relevant Aβ(1–42) amyloid fibril. Proc. Natl. Acad. Sci. USA.

[23] Guerrero-Ferreira R., Nicholas M.I., Mona T.D., Ringler P., Lauer M.E., Riek R., Britschgi M., Stahlberg H. (2018). Cryo-EM structure of alpha-synuclein fibrils. bioRxiv.

[24] Pan K.M., Baldwin M., Nguyen J., Gasset M., Serban A., Groth D., Mehlhorn I., Huang Z., Fletterick R.J., Cohen F.E. (1993). Conversion of α-Helices into β-Sheets Features in the Formation of the Scrapie Prion Proteins. Proc. Natl. Acad. Sci. USA.

[25] Wan W., Wille H., Stöhr J., Kendall A., Bian W., McDonald M., Tiggelaar S., Watts J.C., Prusiner S.B., Stubbs G. (2015). Structural Studies of Truncated Forms of the Prion Protein PrP. Biophys. J..

[26] Vázquez-Fernández E., Vos M.R., Afanasyev P., Cebey L., Sevillano A.M., Vidal E., Rosa I., Renault L., Ramos A., Peters P.J. (2016). The Structural Architecture of an Infectious Mammalian Prion Using Electron Cryomicroscopy. PLoS Pathog..

[27] Smirnovas V., Baron G.S., Offerdahl D.K., Raymond G.J., Caughey B., Surewicz W.K. (2011). Structural Organization of Brain-Derived Mammalian Prions Examined by Hydrogen-Deuterium Exchange. Nat. Struct. Mol. Biol..

[28] Silva C.J., Vázquez-Fernández E., Onisko B., Requena J.R. (2015). Proteinase K and the Structure of PrPSc: The Good, the Bad and the Ugly. Virus Res..

[29] Tsemekhman K., Goldschmidt L., Eisenberg D.S., Baker D. (2007). Cooperative Hydrogen Bonding in Amyloid Formation. Protein Sci..

[30] Kobe B., Kajava A.V. (2000). When Protein Folding Is Simplified to Protein Coiling: The Continuum of Solenoid Protein Structures. Trends Biochem. Sci..

[31] Wasmer C., Lange A., Melckebeke H.V., Siemer A.B., Riek R., Meier B.H. (2008). Amyloid Fibrils of the HET-s(218–289) Prion Form a B Solenoid with a Triangular Hydrophobic Core. Science.

[32] Yoder M.D., Jurnak F. (1995). Protein motifs. 3. The parallel beta helix and other coiled folds. FASEB J..

[33] Coustou V., Deleu C., Saupe S., Begueret J. (1997). The Protein Product of the Het-S Heterokaryon Incompatibility Gene of the Fungus Podospora Anserina Behaves as a Prion Analog. Proc. Natl. Acad. Sci. USA.

[34] Wickner R.B. (1997). A New Prion Controls Fungal Cell Fusion Incompatibility. Proc. Natl. Acad. Sci. USA.

[35] Dos Reis S., Coulary-Salin B., Forge V., Lascu I., Bégueret J., Saupe S.J. (2002). The HET-S Prion Protein of the Filamentous Fungus Podospora Anserina Aggregates in Vitro into Amyloid-like Fibrils. J. Biol. Chem..

[36] Melckebeke V., Wasmer C., Lange A., Ab E., Loquet A., Böckmann A., Meier B.H. (2010). Atomic-Resolution Three-Dimensional Structure of HET-S(218–289) Amyloid Fibrils by Solid-State NMR. J. Am. Chem. Soc..

[37] Ritter C., Maddelein M.-L., Siemer A.B., Lührs T., Ernst M., Meier B.H., Saupe S.J., Riek R. (2005). Correlation of Structural Elements and Infectivity of the HET-S Prion. Nature.

[38] Balguerie A., Dos Reis S., Ritter C., Chaignepain S., Coulary-Salin B., Forge V., Bathany K., Lascu I., Schmitter J.M., Riek R. (2003). Domain Organization and Structure-Function Relationship of the HET-S Prion Protein of Podospora Anserina. EMBO J..

[39] Greenwald J., Buhtz C., Ritter C., Kwiatkowski W., Choe S., Maddelein M.-L., Ness F., Cescau S., Soragni A., Leitz D. (2010). The Mechanism of Prion Inhibition by HET-S. Mol. Cell.

[40] Liebman S.W., Chernoff Y.O. (2012). Prions in Yeast. Genetics.

[41] Mizuno N., Baxa U., Steven A.C. (2011). Structural Dependence of HET-S Amyloid Fibril Infectivity Assessed by Cryoelectron Microscopy. Proc. Natl. Acad. Sci. USA.

[42] Wan W., Wille H., Stöhr J., Baxa U., Prusiner S.B., Stubbs G. (2012). Degradation of fungal prion HET-s(218–289) induces formation of a generic amyloid fold. Biophys. J..

[43] Wan W., Stubbs G. (2014). Fungal Prion HET-S as a Model for Structural Complexity and Self-Propagation in Prions. Proc. Natl. Acad. Sci. USA.

[44] Kajava A.V., Steven A.C. (2006). β-Rolls, β-Helices, and Other β-Solenoid Proteins. Adv. Protein Chem..

[45] Kajava A.V., Steven A.C. (2006). The Turn of the Screw: Variations of the Abundant β-Solenoid Motif in Passenger Domains of Type V Secretory Proteins. J. Struct. Biol..

[46] Kajava A.V., Baxa U., Steven A.C. (2010). Beta Arcades: Recurring Motifs in Naturally Occurring and Disease-Related Amyloid Fibrils. FASEB J..

[47] Hennetin J., Jullian B., Steven A.C., Kajava A.V. (2006). Standard Conformations of β-Arches in β-Solenoid Proteins. J. Mol. Biol..

[48] Jenkins J., Pickersgill R. (2001). The Architecture of Parallel β-Helices and Related Folds. Prog. Biophys. Mol. Biol..

[49] Yoder M.D., Lietzke S.E., Jurnak F. (1993). Unusual Structural Features in the Parallel β-Helix in Pectate Lyases. Structure.

[50] Henrissat B., Heffron S.E., Yoder M.D., Lietzke S.E., Jurnak F. (1995). Functional Implications of Structure-Based Sequence Alignment of Proteins in the Extracellular Pectate Lyase Superfamily. Plant Physiol..

[51] Bryan A.W., Starner-Kreinbrink J.L., Hosur R., Clark P.L., Berger B. (2011). Structure-Based Prediction Reveals Capping Motifs That Inhibit β-Helix Aggregation. Proc. Natl. Acad. Sci. USA.

[52] Kondo H., Hanada Y., Sugimoto H., Hoshino T., Garnham C.P., Davies P.L., Tsuda S. (2012). Ice-Binding Site of Snow Mold Fungus Antifreeze Protein Deviates from Structural Regularity and High Conservation. Proc. Natl. Acad. Sci. USA.

[53] Nelson R., Sawaya M.R., Balbirnie M., Madsen A.Ø., Riekel C., Grothe R., Eisenberg D. (2005). Structure of the Cross-β Spine of Amyloid-like Fibrils. Nature.

[54] Sawaya M.R., Sambashivan S., Nelson R., Ivanova M.I., Sievers S.A., Apostol M.I., Thompson M.J., Balbirnie M., Wiltzius J.J.W., McFarlane H.T. (2007). Atomic Structures of Amyloid Cross-β Spines Reveal Varied Steric Zippers. Nature.

[55] Riek R., Eisenberg D.S. (2016). The Activities of Amyloids from a Structural Perspective. Nature.

[56] Wiltzius J.J.W., Landau M., Nelson R., Sawaya M.R., Apostol M.I., Goldschmidt L., Soriaga A.B., Cascio D., Rajashankar K., Eisenberg D. (2009). Molecular Mechanisms for Protein-Encoded Inheritance. Nat. Struct. Mol. Biol..

[57] Goedert M., Spillantini M.G., Jakes R., Rutherford D., Crowther R.A. (1989). Multiple Isoforms of Human Microtubule-Associated Protein Tau: Sequences and Localization in Neurofibrillary Tangles of Alzheimer’s disease. Neuron.

[58] Mandelkow E.M., Mandelkow E. (1998). Tau in Alzheimer’s Disease. Trends Cell Biol..

[59] Crowther R.A. (1991). Straight and Paired Helical Filaments in Alzheimer Disease Have a Common Structural Unit. Proc. Natl. Acad. Sci. USA.

[60] Šimić G., Babić Leko M., Wray S., Harrington C., Delalle I., Jovanov-Milošević N., Bažadona D., Buée L., de Silva R., Di Giovanni G. (2016). Tau Protein Hyperphosphorylation and Aggregation in Alzheimer’s Disease and Other Tauopathies, and Possible Neuroprotective Strategies. Biomolecules.

[61] Kirschner D.A., Abraham C., Selkoe D.J. (1986). X-ray diffraction from intraneuronal paired helical filaments and extraneuronal amyloid fibers in Alzheimer disease indicates cross-beta conformation. Proc. Natl. Acad. Sci. USA.

[62] Schweers O., Schönbrunn-Hanebeck E., Marx A., Mandelkow E. (1994). Structural Studies of Tau-Protein and Alzheimer Paired Helical Filaments Show No Evidence for Beta-Structure. J. Biol. Chem..

[63] von Bergen M., Barghorn S., Biernat J., Mandelkow E.M., Mandelkow E. (2005). Tau aggregation is driven by a transition from random coil to beta sheet structure. Biochim. Biophys. Acta.

[64] Margittai M., Langen R. (2004). Template-Assisted Filament Growth by Parallel Stacking of Tau. Proc. Natl. Acad. Sci. USA.

[65] Baxa U. (2008). Structural Basis of Infectious and Non-Infectious Amyloids. Curr. Alzheimer Res..

[66] Lührs T., Ritter C., Adrian M., Riek-Loher D., Bohrmann B., Döbeli H., Schubert D., Riek R. (2005). 3D structure of Alzheimer’s amyloid-beta(1–42) fibrils. Proc. Natl. Acad. Sci. USA.

[67] Schmidt M., Rohou A., Lasker K., Yadav J.K., Schiene-Fischer C., Fändrich M., Grigorieff N. (2015). Peptide Dimer Structure in an Aβ(1–42) Fibril Visualized with Cryo-EM. Proc. Natl. Acad. Sci. USA.

[68] Riek R. (2017). The Three-Dimensional Structures of Amyloids. Cold Spring Harb. Perspect. Biol..

[69] Goldsbury C.S., Wirtz S., Müller S.A., Sunderji S., Wicki P., Aebi U., Frey P. (2000). Studies on the in Vitro Assembly of Aβ1-40: Implications for the Search for a Beta Fibril Formation Inhibitors. J. Struct. Biol..

[70] Sachse C., Xu C., Wieligmann K., Diekmann S., Grigorieff N., Fändrich M. (2006). Quaternary Structure of a Mature Amyloid Fibril from Alzheimer’s Aβ(1–40) Peptide. J. Mol. Biol..

[71] Rodriguez J.A., Ivanova M.I., Sawaya M.R., Cascio D., Reyes F.E., Shi D., Sangwan S., Guenther E.L., Johnson L.M., Zhang M. (2015). Structure of the Toxic Core of α-Synuclein from Invisible Crystals. Nature.

[72] Emamzadeh F.N. (2016). Alpha-Synuclein Structure, Functions, and Interactions. J. Res. Med. Sci..

[73] Spillantini M.G., Schmidt M.L., Lee V.M.-Y., Trojanowski J.Q., Jakes R., Goedert M. (1997). Alpha-Synuclein in Lewy Bodies. Nature.

[74] Roeters S.J., Iyer A., Pletikapić G., Kogan V., Subramaniam V., Woutersen S. (2017). Evidence for Intramolecular Antiparallel Beta-Sheet Structure in Alpha-Synuclein Fibrils from a Combination of Two-Dimensional Infrared Spectroscopy and Atomic Force Microscopy. Sci. Rep..

[75] Hutchinson E.G., Thornton J.M. (1993). The Greek Key Motif: Extraction, Classification and Analysis. Protein Eng..

[76] Giasson B.I., Murray I.V.J., Trojanowski J.Q., Lee V.M.Y. (2001). A Hydrophobic Stretch of 12 Amino Acid Residues in the Middle of α-Synuclein Is Essential for Filament Assembly. J. Biol. Chem..

[77] Tzotzos S., Doig A.J. (2010). Amyloidogenic Sequences in Native Protein Structures. Protein Sci..

[78] Zweckstetter M., Requena J.R., Wille H. (2017). Elucidating the structure of an infectious protein. PLoS Pathog..

[79] Peralta M.D.R., Karsai A., Ngo A., Sierra C., Fong K.T., Hayre N.R., Mirzaee N., Ravikumar K.M., Kluber A.J., Chen X. (2015). Engineering Amyloid Fibrils from β-Solenoid Proteins for Biomaterials Applications. ACS Nano.

[80] Helmus J.J., Surewicz K., Surewicz W.K., Jaroniec C.P. (2010). Conformational flexibility of Y145Stop human prion protein amyloid fibrils probed by solid-state nuclear magnetic resonance spectroscopy. J. Am. Chem. Soc..

[81] Helmus J.J., Surewicz K., Apostol M.I., Surewicz W.K., Jaroniec C.P. (2011). Intermolecular alignment in Y145Stop human prion protein amyloid fibrils probed by solid-state NMR spectroscopy. J. Am. Chem. Soc..

